# Naked Oat and *Fusarium culmorum* (W.G.Sm.) Sacc. Responses to Growth Regulator Effects

**DOI:** 10.3390/pathogens12081051

**Published:** 2023-08-17

**Authors:** Sulukhan K. Temirbekova, Oksana B. Polivanova, Irina I. Sardarova, Sholpan O. Bastaubaeva, Elena A. Kalashnikova, Marat Sh. Begeulov, Mukhtar Zh. Ashirbekov, Yuliya V. Afanasyeva, Natalya S. Zhemchuzhina, Natalya E. Ionova, Natalia V. Statsyuk, Rima N. Kirakosyan, Abdulrahman Saleh

**Affiliations:** 1 All-Russian Research Institute of Phytopathology, Bolshye Vyazemy 143050, Russia; irina.sardarova@gmail.com (I.I.S.); zhemch@mail.ru (N.S.Z.); nataafg@gmail.com (N.V.S.); 2 Department of Biotechnology, Russian State Agrarian University—Moscow Timiryazev Agricultural Academy, Timiryazevskaya Street 49, Moscow 127434, Russia; polivanovaoks@gmail.com (O.B.P.); kalash0407@mail.ru (E.A.K.); mbegeulow@rgau-msha.ru (M.S.B.); r.kirakosyan@rgau-msha.ru (R.N.K.); abdulrahman1996nez@gmail.com (A.S.); 3 Kazakh Scientific Research Institute of Agriculture and Plant Growing, Almalybakvillage 021601, Kazakhstan; sh.bastaubaeva@mail.ru; 4 Department of Agronomy and Forestry, Faculty of Agronomy, Manash Kozybayev North Kazakhstan University, 86 Pushkin St., Petropavlovsk 150000, Kazakhstan; mukhtar_agro@mail.ru; 5 Federal Horticultural Center for Breeding, Agrotechnology and Nursery, Zagoryevskaya Street 4, Moscow 115598, Russia; yuliya_afanaseva_90@bk.ru; 6 Biotechnology and Pharmacology, Department of Biochemistry, Institute of Fundamental Medicine and Biology, Kazan Federal University, 18 Kremlyovskaya St., Kazan 420008, Russia; alekta-meg@list.ru

**Keywords:** biotic stress, antioxidant protection, in vitro, in vivo, flowering, biochemical parameters, breeding lines

## Abstract

The antioxidant defense system can be stimulated by growth regulators in plants when they are under stress, such as exposure to pathogens. There are a lot of natural growth regulators on the market, but no research has been carried out yet to determine how effective they are. This field and laboratory study examines the impact of two commonly used Russian growth regulators, Crezacin and Zircon, along with artificial infection with *Fusarium culmorum* on the antioxidant system of naked oat. The results show that, compared to the control, Crezacin-treated plants had higher contents of low molecular weight fructose and nonenzymatic antioxidants like proline, phenolic compounds, and flavonoids. Zircon-treated plants had a lower content of proline, carbohydrates, and lower total antioxidant activity than the control plants. The obtained data show that Crezacin treatment mainly affected nonenzymatic systems of the antioxidant defense. This treatment was more successful than the Zircon application, which did not show any appreciable effectiveness and was typically associated with an improvement in oat productivity. The treatment with growth regulators and a fungal suspension performed at the flowering phase provided the best effect on the biochemical parameters and productivity of naked oats. Moreover, oat treatment with the pathogen promoted the reproductive capabilities of the plants, while growth regulators helped in avoiding infectious stress.

## 1. Introduction

The production of reactive oxygen species (ROS) is a phenomenon typically associated with plant cellular metabolism. According to Das et al. [1], ROS include non-free radical molecules like hydrogen peroxide (H_2_O_2_) and singlet oxygen (O_2_), as well as free radicals like the superoxide anion and hydroxyl radical. In plants, ROS are also used for signaling. Nevertheless, due to their chemical nature, ROS can have damaging effects on various biological components including protein inactivation, membrane rupture, and DNA damage. Like other aerobic species, plants use effective ROS scavenging mechanisms, which include both nonenzymatic and enzymatic chemical antioxidant systems. In plants subjected to abiotic and biotic stressors, enzymes such as superoxide dismutase, catalase, and peroxidase are crucial for maintaining redox balance and defense response [2,3,4,5]. The main nonenzymatic plant antioxidants are ascorbic acid, tocopherol, carotenoids, and various phenolic compounds [6].

Hyperproduction of ROS derivatives, which typically occurs under diverse biotic and abiotic conditions, may lead to the induction of defensive mechanisms against ROS. ROS production is a part of a plant’s response to pathogen attacks. The role of ROS during pathogen penetration into a cell is related to their ability to directly strengthen the host cell by cross-linking glycoproteins in the membrane [7]. It is also obvious that ROS are important signals in mediating the activation of protective genes [8].

The enhanced biosynthesis of phenolic compounds and other nonenzymatic antioxidants in plants has been observed in response to pathogen attack and excessive ROS production [9,10,11,12]. The synthesis of specific phenolic compounds can be caused by a contact between the pathogen and the host. For example, secretion of de novo synthesized trans-cinnamic acid occurs during *Fusarium* infection of barley roots [13]. Antioxidant phenols presenting in cereal grains modulate mycotoxin production in *F. graminearum*. Some compounds were found to increase toxin production, while some decreased it via structure-dependent signals [14]. Thus, phenolic compounds are actively involved in the interaction between plants and fungal pathogens as signaling molecules. Although phenolic compounds are more commonly associated with plant response to bacterial infections and insects, they also possess antifungal activity. Cinnamic acid, benzoic acid, salicylic acid, thymol, and dihydroxybenzaldehydes taken at a concentration of 5 mM can inhibit the growth of some *Candida* species and *C. neoformans* by >90%. As expected, clinical antifungal drugs are effective at much lower concentrations. However, combinations of phenols and antifungals demonstrate greater efficacy [15].

During stress, plants also produce a variety of osmoprotectants. Under osmotic stress, proline aggregation is observed in a number of higher plant species. Proline also provides the scavenging of free radicals and the stability of proteins and membranes. It may also increase the activity of several enzymes [16,17].

Soluble sugars play a key role in plant development and metabolism; therefore, their content changes significantly during infection and plant interaction with pathogens. Soluble sugars primarily serve as a carbon source for a pathogen in host plant cells [18,19]. According to some data, sucrose induces defense mechanisms in infected plant cells. Hexose increases the production of peroxidases and proteins directly related to pathogenesis via the hexokinase signaling pathway [20,21]. Being compounds with a higher osmotic potential, soluble sugars limit the spreading of infection in the plant. Moreover, they isolate healthy cells from infected ones and protect them from water loss [22].

Oat (*Avena sativa* L.) is widely cultivated as a grain and fodder crop on a global scale, especially in northern Europe. Oat is of great importance for human and animal nutrition, since it is characterized by high contents of starch, protein, and a balanced amino acid composition [23]. It also contains dietary fibers, unsaturated fatty acids, and phytonutrients, making it beneficial in maintaining human health.

Oat is highly susceptible to fungal infections including *Fusarium* rot, which widely affects cereals in northern countries. Most often, *Fusarium* rot is associated with such pathogens as *Fusarium graminearum*, *F. culmorum*, *F. avenaceum,* and *F. poae* [24]. The majority of studies on *Fusarium*-caused diseases of cereals have primarily been conducted on barley and wheat as the most significant food crops. In recent years, the scientific interest in the *Fusarium* diseases of oat arose due to increased demand for a high-quality grain of this crop, which is used both in the feed and food industries. *Fusarium* rot of cereals, including oat, causes significant economic loss, as well as various human and animal diseases [25]. In this regard, there is a need for safe and effective approaches to controlling the spread of *Fusarium* rot.

Plant growth regulators are important components of the agricultural industry, as they have a great potential for the plant productivity increase. They are simple in use and often inexpensive. In addition, as they are not species-specific, plant growth regulators can be used on many crops [26]. Today, many of commercially available growth regulators are derived from natural sources (e.g., kelp extract, humic substances, or protein hydrolysates) that make them environmentally friendly. A commercial growth regulator Zircon represents a mix of hydroxycinnamic acids. According to its manufacturer, Zircon increases seed germination, accelerates plant flowering, growth and development, and provides a yield increase by 35–50%. The stimulating effect of cinnamic acids on plant growth and development is well known. For example, the treatment of Arabidopsis roots with low concentrations of cis-cinnamic acid stimulated cell growth and division [26]. Ferulic and caffeic acids were also shown to possess growth-stimulating properties due to their auxin-like action [27].

Tris-2(hydroxyethyl)ammonium 2-methylphenoxyacetate (Crezacin, Trecresan) was synthesized in the 1990s at the Favorsky Institute of Chemistry (Irkutsk, Russia). According to the manufacturer’s statement, this compound is an affordable, effective, low-toxic, and environmentally friendly growth stimulator able to improve the productivity of agricultural plants [28].

In relation to the environmental safety, plant growth regulators obtained from natural sources have an advantage over those produced by chemical synthesis. Their use may be promising for increasing crop productivity as well as for activating disease resistance mechanisms. Crezacin and Zircon growth regulators are widely presented in the Russian market. However, evidence for the effectiveness of these commercially available preparations is insufficient. The aim of this study was to investigate the effect of the Crezacin and Zircon growth regulators combined with the artificial infection with *F. culmorum* on antioxidant defense systems and biochemical parameters of naked and chaffy oat.

## 2. Materials and Methods

The study was carried out in 2019–2020 at the experimental field of the All-Russian Research Institute of Phytopathology (the assessment of biochemical parameters of the best naked oat lines is currently underway). The objects of the study were chaffy oat varieties “Bulany” and “Ulov” (Federal Research Center “Nemchinovka”, Moscow region, Russia) and naked oat line no. 7 (MOVIR Institute of Plant Production, Moscow region, Russia). Thirteen lines derived from collected samples of naked oat, including k-14987 Lawrel, k-11594, k-15084 Lemont (all from USA), k-14530 OA504-6, and k-14919 ACGWEN (all from Canada), were assigned and improved due to an extensive study performed in 2008–2016 [29]. Field trials arranged in 2017–2019 in Kazakhstan (Kazakh Institute of Agriculture and Plant Growing, KAZiZR) and Russia (Moscow region) showed the best results for line nos. 1, 4, 7, and 10. These lines were isolated from the North American group of *Avena sativa* L., which included varieties *inermis* and *chinensis*. Line no. 1 (*chinensis*) Bacha 1 is being tested by Dr. Ion Tonca (Institute of Plant Industry, Bucharest, Romania) since 2016; line nos. 4 and 7 (*inermis*), isolated from US samples, are being tested in the Moscow region. Samples from Canada included isolated lines 10 and 13. 

The experimental field was characterized by thick loamy, sod-podzol soils with a well-defined podzol horizon. The underlying rock was a bog loam, which weakened erosion processes. A humus content determined according to Tyurin was 2.5–3.2%; the contents of mobile P_2_O_5_ and exchangeable K_2_O were determined according to Kirsanov and Maslov, respectively, and reached 12–18 and 15–23 mg per 100 g of soil, respectively. A pH level of a salt extract was 5.6–7.0. The climate was moderately humid and temperate continental with the average annual rainfall of 450–800 mm. A hydrothermal coefficient was 1.3–1.1. All field experiments were carried out in triplicate. The land treatment corresponded to that common for this region. The level of internal contamination of oat grain with *Fusarium culmorum* was determined according to Bulay et al. [30]. 

The year 2019 was moderately wet with the total precipitation during the growing season equal to 212 mm (the average long-term standard value for the growing season is 264 mm). The average temperature during a vegetation season was +17.0 °C (the average annual temperature is +15.1 °C). In 2020, the amount of precipitation during the growing season was 343 mm, and the average temperature was +20.7 °C. The hydrothermal coefficient was 1.1–1.3.

**Figure 1:**
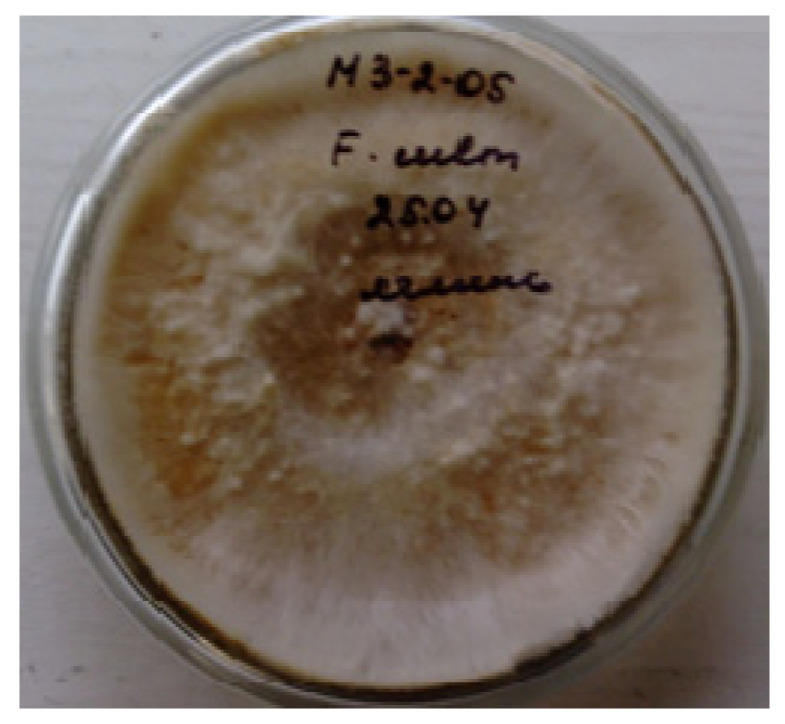
Live culture of the *Fusarium culmorum* strain M-2-3.

The artificial infection of plants was carried out using a *Fusarium culmorum* (W.G.Sm) Sacc. strain M-2-3. The strain was isolated in 2005 from a barley plant grown in the Moscow region (Figure 1). Strain isolation and identification were carried out at the Mycology Laboratory of the All-Russian Research Institute of Phytopathology. A pure culture of the pathogen was stored for a long time at +4 °C. To restore functional activity of the pathogen, the fungus was passed through 5 variants of nutrient media recommended for growing of the *Fusarium culmorum*: potato agar, Chapek medium, etc. The pure culture of the pathogen was reproduced using a hormone-free Murashige and Scoog agar medium [31]. The culture transfer was carried out after a 5–7-day incubation under sterile conditions. The fugus was identified using a key proposed by V.I. Bilay [30]. The microscopic examination of the fungus was carried out at a 400–600× magnification using a “crushed drop” method.

The treatment of plants with the fungal suspension and growth regulators was carried out during the heading, early flowering, and full flowering phases in dry and calm weather. Crezacin and Zircon [26,27,28] concentrations were 30 and 10 mg/L, respectively, which corresponded to the manufacturer’s recommendations. Untreated plants were used as the control. The effect of treatment was evaluated using such parameters as the 1000 kernel weight and the yield (g/m^2^). The level of grain infection with *Fusarium* fungi was assessed by both visual evaluation and determination of the internal contamination level. No PCR diagnostics were used.

Plants grown in the field were treated three times; the plot size for each replicate was 2 m^2^. Crops were divided into strips, and each strip was treated with a suspension of the pathogen as well as with Crezacin and Zircon, according to the experimental scheme presented in the results of the studies.

The grain was harvested using a Sampo-130 combine harvester. 

Each replication produced 170–250 g of grain. For laboratory studies, grain samples were taken from each replication. The experiments were carried out in three biological and five analytical replicates. The control variant included grain obtained from untreated plants.

Grains were germinated in order to determine some biochemical characteristics. Three samples were evaluated: the control sample and samples treated with fungal suspension or growth regulators at the full flowering stage. This choice was conditioned by the highest productivity of these samples. To produce seedlings, grains were germinated on Petri plates for 7 days. 

Plant material was freeze-dried for 24 hat −80 °C and 7 mTorr, then ground in a mortar and extracted with 80% ethanol at a 1:100 ratio. The resulting extracts were freeze-dried and used for analysis by extracting biologically active substances (BAS) with a suitable solvent.

The content of free proline was determined using an acid ninhydrin reagent (1.25 g ninhydrate + 30 mL glacial acetic acid + 20 mL 6M H_3_PO_4_). A 200-mg sample was supplemented with 5 mL of distilled water and kept for 10 min in a water bath at 100 °C. Then, 2 mL of glacial acetic acid and 2 mL of a ninhydrin reagent were mixed in a clean test tube, and 2 mL of the prepared extract was added. The sample was incubated for 20 min in a water bath at 100 °C, then quickly cooled to room temperature using cold water or ice, and the optical density of the reaction product was spectrophotometrically measured at 520 nm. The blank solution contained 2 mL of distilled water, 2 mL of glacial acetic acid and 2 mL of ninhydrin reagent. Analytically pure proline (Sigma-Aldrich, St. Louis, MO, USA) was used to build a calibration curve [32].

The content of low-molecular fructose was determined by the following way: 0.5 µL of 25% NaOH was added to 99.5 µL of the sample extract and after 10 min incubation centrifuged at 3000 rpm for 3 min. The supernatant (50 μL) was mixed with 1 mL of the resorcinol reagent (2 mg/mL of resorcinol mixed with 96% ethanol and concentrated HCl at a 1:1 ratio), incubated in a water bath at 80 °C for 30 min, and supplemented with water up to a final volume of 10 mL. The optical density of the final solution was measured at 480 nm.

To determine the content of phenolic compounds, 200 µL of the Folin–Ciocalteu reagent (10%) was thoroughly mixed with 100 µL of a sample. Then, 800 µL of a 700 mM Na_2_CO_3_ solution was added, and the mix was incubated for 2 h at room temperature. The optical density was measured at 765 nm. A calibration curve (absorbance vs. concentration) was built using standard solutions of gallic acid [33].

The content of total flavonoids was measured using the reaction of complexation with aluminum chloride. A sample extract (0.1 mL) was adjusted to 1 mL with 96% ethanol, and supplemented with 0.5 mL of a 1.2% ethanol solution of aluminum chloride and 0.5 mL of a 120 mM aqueous solution of potassium acetate. After a 30 min incubation, the optical density of a mixture was spectrophotometrically measured at 425 nm. A blank solution contained 100 µL of 80% ethanol + 900 µL of 96% ethanol + 0.5 mL of 1.2% ethanol solution of aluminum chloride + 0.5 mL of 120 mM aqueous potassium acetate solution [34].

The antioxidant activity of sample extracts was measured according to Oyaizu [35]. Various concentrations of extracts (200–1200 ppm) were dissolved in 1 mL of distilled water, then 2.5 mL of phosphate buffer (0.2 M, pH 6.6) and 2.5 mL of 1% K_3_[Fe(CN)_6_] were added. The mixture was incubated in a water bath for 20 min at 50 °C. Then, 2.5 mL of 10% trichloroacetic acid was added and centrifuged for 10 min at 3000 rpm. A supernatant (2.5 mL) was mixed with 2.5 mL of water and 0.5 mL of 0.1% ferric chloride, and the optical density of the resulting solution was measured at 700 nm. The antioxidant activity of various dilutions of 2.5 mg/mL ascorbic acid (200–1200 ppm) was determined by the same method followed by the calculation of the number of antioxidants in the extracts.

Catalase activity was evaluated according to Goth [36] using the following solution: 1 mL 65 µM H_2_O_2_ in 60 mM K-Na-phosphate buffer (substrate). The sample extract (200 μL) was added to the H_2_O_2_ solution, and after 60 s of incubation, the reaction was terminated by adding 1 mL of 32.4 mM ammonium molybdate ((NH_4_)_6_ Mo_7_O_24_ · 4H_2_O), and the sample adsorption at 410 nm was determined (sample A). To calculate the activity of the enzyme, the following mixtures were measured at the same wavelength:

1. First, 1 mL of ammonium molybdate and 200 μL of the sample extract were added to 1 mL of a substrate, and the adsorption of the mixture (A1) was determined.
2. Next, 1 mL of the substrate was supplemented with 1 mL of ammonium molybdate and 200 μL of 60 mM K-Na-phosphate buffer, and the adsorption of the mixture (A2) was determined.
3. Finally, 1.2 mL of 60 mM K-Na-phosphate buffer was supplemented with 1 mL of ammonium molybdate followed by the adsorption measurement (A3).

The catalase activity calculated according to the following formula:

Catalase activity (kU/L) = ((A − A1) · 271)/(A2 − A3).

In addition, a protein content in the sample preparation was quantified using a qualitative reaction with Coomassie Brilliant Blue G250 [37]. The obtained values were converted to nCat/g of protein.

Ascorbate peroxidase activity was determined according to Nakano and Asada [38]. The working solution included 1.5 mL of 50 mM K-Na-phosphate buffer (pH 7.0), 500 µL of 2.5 mM ascorbic acid solution; 500 µL of 1 mM H_2_O_2_ solution, and 100 µL of 0. mM EDTA solution. The optical density of the solution was measured every 10 s at 290 nm within 2–3 min after addition of the sample extract (400 μL). A blank solution contained 1.5 mL of 50 mM K-Na-phosphate buffer (pH 7.0), 1 mL of distilled water, 400 µL of the sample extract, and 300 µL of 0.5 mM EDTA. Each measurement was carried out in triplicate. In addition, a protein content in the sample preparation was quantified using a qualitative reaction with Coomassie Brilliant Blue G250. The obtained values were converted to nCat/g of protein.

Peroxidase activity was evaluated according to Popov and Neikovska [39]. The working solution included 1 mL of 0.2 M Na-acetate buffer (pH 4.9); 500 µL of 4 µM indigo carmine solution, and 500 µL of 0.03 M H_2_O_2_ solution. After addition of a sample extract (500 μL), the optical density of the resulting solution at 610 nm was measured every 15 s within 2 min. A blank solution contained an equal volume of distilled water instead of the sample extract. In addition, the protein content in the sample preparation was quantified using a qualitative reaction with Coomassie Brilliant Blue G250. The obtained values were converted to nCat/g of protein.

All measurements of both experimental and control samples were arranged in three replications. 

The content of protein/nitrogen was determined by a Kjeldahl method with some modifications; the protein was calculated using a conversion factor of 5.7. The weight of the used flour sample was 0.3 g [40,41]. The analysis was performed using a Kjeltec 2200 semiautomatic analyzer (FOSS, Hilleroed, Denmark) equipped with an automatic distillation unit. The oil content was determined by the weight of a dry fat-free residue in a Soxhlet apparatus using petroleum ether as a solvent (t_0_bp −40–700 °C). The starch content was determined by a polarimetric method according to Evers using a 0.2 g flour sample [40,42].

The obtained results were expressed as the mean value ± confidence interval for three replicates. Analysis of variance (ANOVA) was used to determine a significant difference (*p* < 0.05). All statistical analyses were performed using Microsoft Excel and Statistica software packages.

## 3. Results

### 3.1. Evaluation of the Yield and 1000 Kernels Weight after Artificial Fusarium Infection and Treatment with Growth Regulators

Fusarium fungi are common pathogens of cereals, causing a wide range of diseases (mainly Fusarium rot) at all plant development stages. Fusarium rot is associated with the development of chalky seeds and a low emergence rate [43]. Food and feed contamination by fusariotoxins can pose serious risks to human and animal health [44]. 

According to the calculated values of the least significant difference, several variants significantly differed from the control (untreated) variant. These variants included oat plants treated at the flowering phase with Zircon + *Fusarium* and Crezacin + *Fusarium;* such treatments significantly increased the weight of 1000 kernels and the yield in both studied oat types.

In relation to the effect of the pathogen on the selected oat types, artificial inoculation of plants did not affect the yield of the chaffy cultivar “Bulany”, while the effect on the naked oat was significant (Table 1).

Fusarium rot rapidly spread in both “control” and “control + infection” variants, demonstrating the importance of continued studies and the necessity to choose the best strategies for preventing the infection spreading. The data shown in Table 1 reliably indicate the effectiveness of the selected growth regulators. Zircon and Crezacin prevented the spreading of infection by 10–20% each, but when combined with the *F. culmorum*, their biological efficiency increased to 40–55% (45% for Zircon + *F. culmorum* and 30% for Crezacin + *F. culmorum*). Unlike Zircon, the use of Crezacin increased the disease resistance of plants (Table 2).

The performed experiment showed that the treatment of two oat crops (chaffy cultivar “Bulany” and naked oat line no. 7) with Crezacin and Zircon improved plant adaptation to the pathogen-induced stress, resulting in a reduced grain infection and thereby contributing to a sufficient yield with a good grain quality. Note that the plant treatment with Crezacin at the full flowering phase was the most effective that agreed with the earlier obtained data [45].

### 3.2. Proline Content Determination 

Accumulation of free proline in plant leaves is a nonspecific plant response to stress caused by abiotic factors (water, temperature, high salinization, air pollution) and infection with fungal pathogens [32].

**Figure 2:**
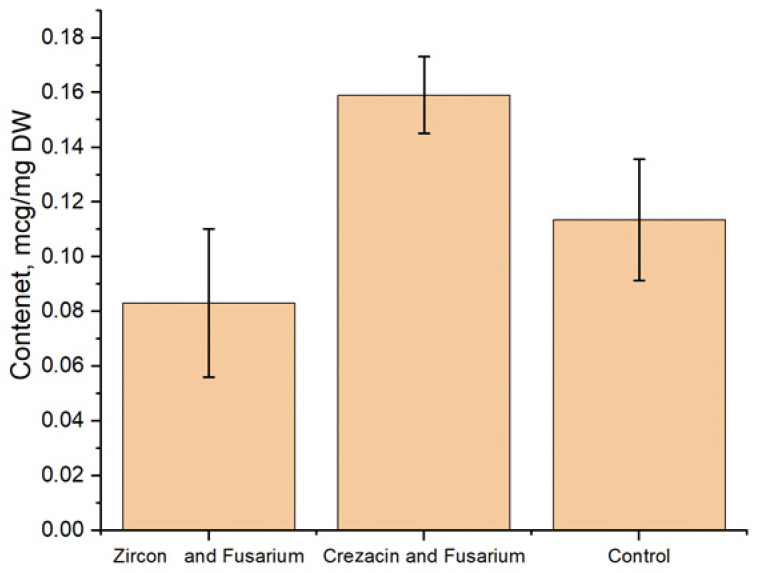
Proline content (µg/mg of dry weight (DW)) in oat seedlings grown from seeds of plants treated with growth regulators and artificially infected with *Fusarium* fungi. The experiments were carried out in three biological and five analytical replicates. Here and below: graphs and diagrams show the arithmetic means and their confidence intervals. Untreated noninfected plants were used as the control.

The results of a free proline content determination in oat seedlings grown from seeds of plants treated with growth regulators are shown in Figure 2.

Compared to the control (no treatment, no infection), the content of proline in the seedlings grown from seeds of plants treated with Crezacin increased to 0.16 µg/mg. The content of free proline in seedlings of the "Zircon" variant was 0.08 μg/mg, which was comparable with the control (0.11 μg/mg). Data showing an increase in the content of free proline and other free amino acids in cereals infected with fungal pathogens are available. After the plant inoculation with *F. culmorum* spores, the accumulation of free amino acids increased by 30–50% compared to the control. The use of biostimulants also increased in the level of proline and other amino acids, though to a lesser extent (3–33%) compared to the control. [46]. 

### 3.3. Determination of the Low-Molecular Fructose Content

**Figure 3:**
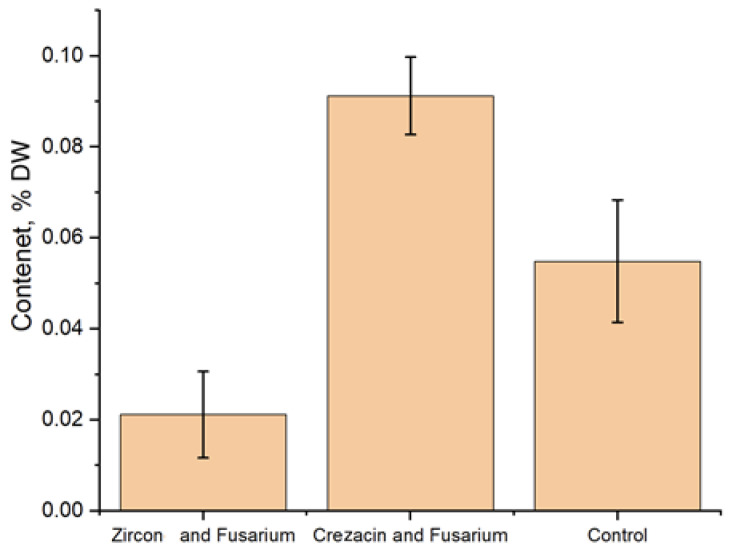
The content of low-molecular fructose (% of dry weight (DW)) in oat plants grown from seeds of plants treated with growth regulators and artificially infected with *Fusarium*. The experiments were arranged in three biological and five analytical replications. Untreated noninfected plants were used as the control.

The content of low-molecular fructose in the studied plant samples is shown in Figure 3. In both variants of treatment, the obtained results significantly differed from the control. In the case of seedlings grown from seeds of Crezacin + *Fusarium*-treated plants, the value of this parameter was 66.3% higher than in the control. In the case of seedlings of the Zircon + *Fusarium* variant, a decrease by 61.6% of the control was observed.

Some studies showed that sugars are involved in the initiation of defense response to abiotic and biotic factors [47]. Sugars are the energy source for intracellular defense responses against pathogens and provide a carbon backbone for the synthesis of secondary metabolites (flavonoids, stilbenes, and lignins) [48]. In addition, sucrose, glucose, fructose, and trehalose represent metabolic signaling molecules inducing the expression of many defense genes in host plant cells [49]. High content of sugars in plant tissues enhances the immune response of plants against fungal pathogens. Sugars function as priming molecules involved in the pathogen-associated molecular pattern (PAMP)-triggered immunity and effector-triggered immunity (ETI) in plants [50,51].

An increase in the sugar content in response to artificial *Fusarium* infection combined with the plant treatment with Crezacin may indicate a stimulatory effect of this growth regulator, including an enhancement of the sugar-mediated immune response. 

### 3.4. Determination of the Content of Phenolic Compounds and Flavonoids 

**Figure 4:**
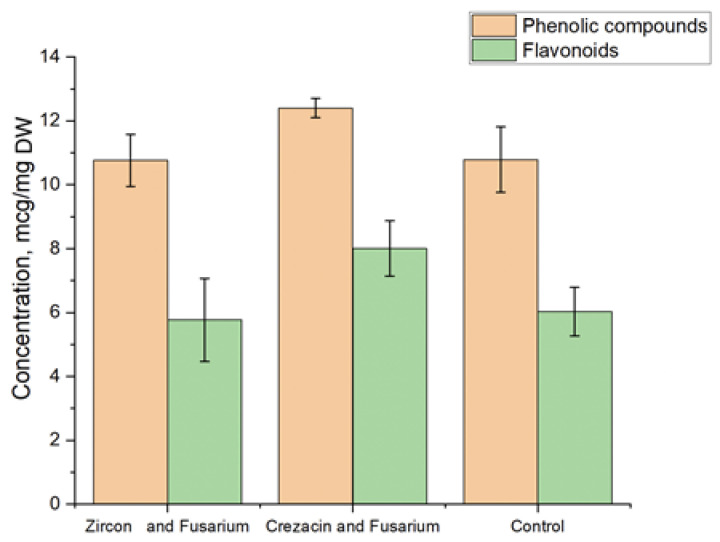
The content of phenols and flavonoids in oat seedlings grown from seeds of plants treated with growth regulators and artificially infected with *Fusarium*. The experiments were arranged in three biological and five analytical replications. Untreated noninfected plants were used as the control.

The content of phenolic compounds and flavonoids in oat seedlings grown from seeds of plants treated with Crezacin and Zircon and artificially infected with *Fusarium* fungi is shown in Figure 4. The treatment with Crezacin resulted in an increase in the content of phenolic compounds and flavonoids compared to the control. No significant differences were observed between the control and Zircon-treated samples.

### 3.5. Antioxidant Activity Determination 

**Figure 5:**
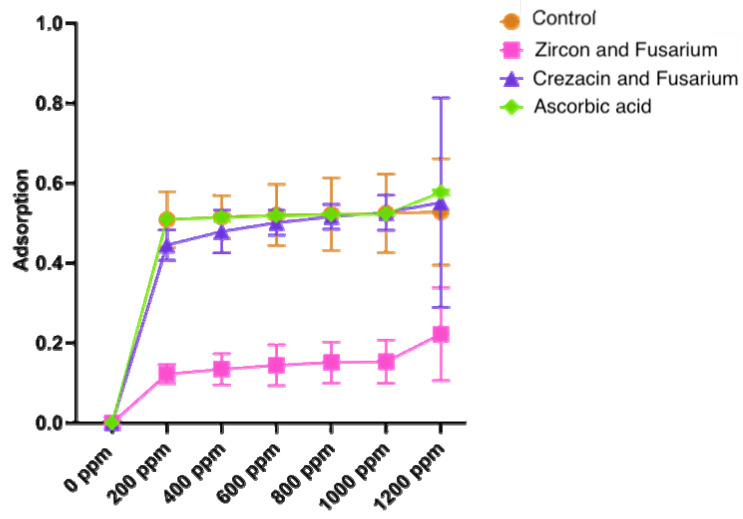
The total ferric reducing power of different variants of oat extracts taken in different concentrations (0–1200 ppm). Ascorbic acid was used as the reference antioxidant. The total ferric reducing power was determined by the transformation of ferric ions (Fe^3+^) to ferrous ions (Fe^2+^). The reducing power estimation was based on the absorbance at 700 nm. The values are expressed as mean ± standard deviation of three replicates. The extract from untreated noninfected plants was used as the control.

The results of the antioxidant activity determination are shown in Figure 5.

The lowest antioxidant activity was shown for a combined treatment with Zircon and *Fusarium*. In this case, the average total antioxidant content (in terms of ascorbic acid) decreased by 50.6% compared to the control.

Irrespectively of the concentration of biologically active substances, the shape of a curve remained the same, which indicated the reliability of the obtained results; the averaged internal correlation coefficient was 0.99. The most statistically differing results were obtained for the extract concentrations of 600 ppm and 800 ppm. No significant difference was observed between the control and Crezacin-treated plants.

### 3.6. Activity of Antioxidant Defense Enzymes

**Figure 6:**
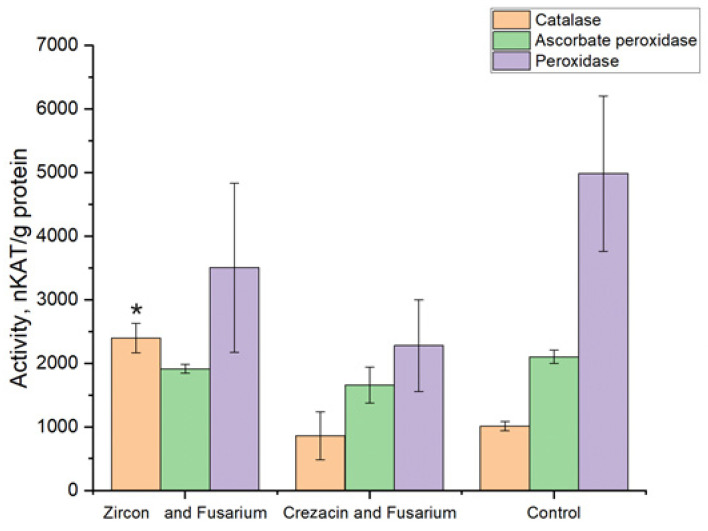
Activity of antioxidant defense enzymes in extracts from oat seedlings grown from seeds of plants treated with growth regulators and artificially infected with *Fusarium* fungi. The experiments were arranged in three biological and five analytical replications. The extract from untreated noninfected plants was used as the control.

Interaction between a plant and a pathogen enhances the activity of CAT, APC, APX, and some other plant enzymes [52]. The results obtained for the activity of key antioxidant defense enzyme in oat seedlings in response to artificial *Fusarium* infection combined with the treatment with Crezacin or Zircon are shown in Figure 6.

An increase in the catalase activity was observed in seedlings obtained from Zircon-treated plants. No significant difference was found between the control seedlings and seedlings obtained from Crezacin-treated plants.

There was no significant difference in the activity of ascorbate peroxidase between experimental variants. A decrease in the peroxidase activity compared to the control values was observed in seedlings obtained from Crezacin-treated plants.

### 3.7. Biochemical Composition of Grain

Naked forms of oat were found to have an advantage over chaffy cultivars in relation to the content of protein, oil, and essential amino acids. In addition, a high resistance to *Fusarium* fungi was revealed for naked oat samples. Therefore, these forms may serve as a breeding reserve for enhancing the oat grain quality in chaffy cultivars grown in Central Russia.

The biochemical composition of grain of naked oat lines is presented in Table 3.

Proteins, polysaccharides and lipids are the main components, on which the nutritional value and technological properties of oat grain depend. Therefore, it was important to study the biochemical features not only for line 7, involved in the field studies, but also for other promising lines of naked oat isolated from the population of naked varieties of American and Canadian origin. In order to evaluate the prospects of their further use in breeding, their biochemical features were examined together with the best line, no. 7, and compared with chaffy oat (Table 3). We have proved that all our lines were characterized by a high protein content (up to 19.7%). The minimum protein content (12.8%) was observed in a chaffy cultivar. Long-term studies of Gubanova and Kozlenko [53] showed that the protein content in chaffy oat cultivars does not exceed 13.0–13.5%.

We have found that all lines of naked oats had a positive correlation between the protein content and the content of essential amino acid lysine per 100 g of grain (R = 0.85; T_fact_ = 3.18; T_theor_ = 2.44). This fact indicates an increase in the lysine content in the grain correlates with an increase in protein content.

The carbohydrate complex has its own important characteristics. Table 3 shows that the maximum content of starch, protein, and lysine for the different studied naked oat lines reached: 57.5, 14.7, and 7.4%, respectively, for line no. 1, (Bacha); 58.1, 16.5, 9.9%, respectively, for line no. 4; 61.0, 16.9, and 6.5%, respectively, for line no. 7; 63.5, 18.3, and 8.1%, respectively, for line no. 10; and 64.0, 19.7, and 9.4%, respectively, for line no. 13. In the case of the chaffy oat variety, the content of above-mentioned nutrients was 51.3, 12.8, and 3.8%, respectively. Note that even line no. 1 (Bacha), which belongs to *Avena sativa* var. *chinensis*, exceeded chaffy oat in terms of these biochemical parameters. 

An important feature determining the nutritional value of oats is the content of lipids. The most effective way to increase the caloric value of grain is the breeding of new lines with an increased lipid content. It was important for us to determine how to work with this parameter not only in line 7, but also in other naked lines listed in Table 3. Our study showed that all our naked lines were characterized not only by a high protein content, but also by a high lipid content (7.0–9.9%); note that the average standard lipid content in oats is 4.8%. Our findings demonstrate that the biochemical differences between naked and chaffy oat lines indicate a high breeding potential for naked oat types with acceptable grain quality.

## 4. Discussion

In the case of such crops as chaffy and naked oats, there are no studies reporting the use of growth regulators for disease prevention. One of the most common and harmful oat diseases is a *Fusarium*-caused disease. *Fusarium* fungi are the primary agents of this disease. Its symptoms are quite variable and include the rotting of seeds in the soil during germination, root rot, seed contamination with mycotoxins, empty ears, worsening of technological properties of grain during storage, and other factors affecting plant productivity [54].

*Fusarium* fungi are the most harmful in the second half of the growing season for the years with a humidity level exceeding 70% and the average daily temperature of 22–25 °C. The damage is expressed via direct (lower grain yield) and indirect (lower quality of a finish product) losses. The affected grain also contains mycotoxins, which, being presented in amounts exceeding specific acceptable limits, may cause harmful effects on both human and animal health [55,56]. For example, they can worsen general immunity, affect internal organs, or even result in a poisoning.

Chemical treatments are frequently used to increase disease resistance of plants, and they still remain important until the discovery of safe alternative solutions [57]. Therefore, from the ecological, practical, and economic perspective, the use of growth regulators is a good way to prevent infections. According to the existing data, some specific concentrations of such compounds influence a plant’s ability to resist infections, because they induce a plant to produce more phenolic compounds [29,58].

Some scientists reported that expression of gene-encoding proteins binding with the cell wall allows plant cells to rapidly respond to the pathogen infection. In response to infection, a rapid strengthening of the cell wall occurs due to the transformation of proline-rich proteins into insoluble forms and their oxidative cross-linking [59]. The accumulation of free proline in plant leaves is a non-specific reaction of plants to stress caused by abiotic factors (humidity, temperature, high soil salinity, air pollution) and infection with fungal pathogens. The treatment with biostimulants also contributes to an increase in the content of proline and other amino acids, though to a lesser extent (3–33%) than in the control [32].

We consider the ability of grain to produce secondary metabolites, which play a protective role in intact plants, reinforcing the effect of Crezacin and Zircon. Such substances include phenolic compounds, the synthesis of which is enhanced under stressful conditions.

The role of phenolic compounds in plant defense systems against pathogens (bacteria, fungi, and viruses) as well as against important abiotic stresses like drought, salinization, and UV radiation is a subject of ongoing studies. The antibacterial and antioxidant characteristics of these substances promote the prevention of plant infections and protect underlying tissues from the harmful effects of reactive oxygen species. Phenol accumulation and activation of the phenylpropanoid biosynthetic pathway were both revealed in response to the environmental stress [60].

Our study included a visual examination of the *Fusarium* infection of oat seeds, which revealed that plants treated with Crezacin were more resistant to infection. Moreover, the yield and the weight of 1000 kernels were also higher in Zircon-treated variants compared to the control. A quantitative analysis of non-enzymatic antioxidants showed that the content of proline and low-molecular fructose was higher in the Crezacin-treated variant. The role of these compounds in the plant protection system is well known. Probably, Crezacin treatment enhances the protective response of oats to the pathogen. At the same time, treatment with Zircon was accompanied by a decrease in the content of proline and low-molecular fructose.

The content of phenolic compounds and flavonoids was also higher in plant seedlings treated with Crezacin. As a result, Crezacin treatment of plants promoted the synthesis of non-enzymatic antioxidants in seedlings grown from plants inoculated with *F. culmorum* under both in vitro and in vivo conditions. The activity of peroxidase in such plants was also reduced, while the activity of catalase and ascorbate peroxidase still remained at the control level. Based on these results, one can conclude that Crezacin mainly affects the enzymatic system of antioxidant protection without significantly affecting the activity of the analyzed enzymes. New plant growth regulators provided a positive effect on the productivity and biochemical parameters of naked oat. Therefore, they should be studied in combination with different biotic and abiotic conditions providing an expanded assessment of their efficiency and practical potential.

Note that, in contrast to chemical fungicides, Crezacin and Zircon growth regulators are natural compounds [61], which are not harmful to either human or animal health, and did not affect the ecological balance of the environment.

## 5. Conclusions

The growth regulators Crezacin and Zircon have been used in this study as an alternative way to control *Fusarium* infection of chaffy and naked oats. The effect of growth regulators combined with the artificial infection with *F. culmorum* on the antioxidant defense systems of oats has been demonstrated.

The influence of these compounds on the mycelial growth of *Fusarium* fungi was shown under in vitro conditions. Both growth regulators were shown to be able to inhibit the development of a pathogen mycelium compared to the control. The performed experiments revealed that the effect of fungal growth inhibition by growth regulators was enhanced during a joint cultivation of the pathogen and plant.

In relation to the oat crop, the authors first revealed that the total content of antioxidants in seedlings and plants cultivated under in vitro and in vivo stress conditions and treated with both Crezacin and Zircon increases in response to biotic (infectious) stress.

The most effective treatment period was the flowering phase. The advantage of naked oat breeding lines over chaffy ones has been shown in terms of the content of proteins, oil, and essential amino acids.

The effect of plant growth regulators on the *Fusarium* inhibition under certain in vivo and in vitro cultivation conditions can be used for preventive treatments of oat crops against infection with a simultaneous reduction of a fungicidal load on the environment.

## Figures and Tables

**Table 1:** Weight of 1000 kernels and the total yield of the “Bulany” oat variety and the naked oat line no. 7 after artificial *Fusarium* infection followed by the plant treatment with the studied preparations (averaged for 2019–2020).

No.	Experimental Variants	Bulany (Chaffy)		Naked Oat Line No. 7	
		1000 Kernel Weight, g	Yield, g/m^2^	1000 Kernel Weight, g	Yield, g/m^2^
1	Control (untreated)	40.7	350	21.1	110
2	Crezacin	42.1	450	20.7	135
3	Zircon	40.0	440	19.8	115
4	Pathogen (*F. culmorum*)	38.9	350	17.3	85
Heading Phase					
5	Zircon + *Fusarium*	38.7	370	23.4	130
6	Crezacin + *Fusarium*	39.2	320	20.8	140
Early Flowering Phase					
7	Zircon + *Fusarium*	37.0	300	21.5	100
8	Crezacin + *Fusarium*	38.0	280	21.9	120
Full Flowering Phase					
9	Zircon + *Fusarium*	43.8	500	22.4	170
10	Crezacin + *Fusarium*	49.3	520	21.4	210
LSD_05_		0.8	20	0.4	11

**Table 2:** Post-harvest internal infection of the grain of chaffy and naked oat with *F. culmorum*, % (averaged for 2019–2020).

No.	Experimental Variants	Presence of *F. culmorum* in the Grain	
		Bulany (Chaffy) Oat	Naked Oat Line No. 7
1	Control (untreated)	88.0	95.0
2	Crezacin	65.0	77.0
3	Zircon	77.0	85.0
4	Pathogen (*F. culmorum*)	90.0	97.0
Heading Phase			
5	Zircon + *Fusarium*	63.79	79.0
6	Crezacin + *Fusarium*	51.69	69.0
Early Flowering Phase			
7	Zircon + *Fusarium*	63.0	79.0
8	Crezacin + *Fusarium*	51.0	69.0
Full Flowering Phase			
9	Zircon + *Fusarium*	45.0	55.0
10	Crezacin + *Fusarium*	30.0	41.0
LSD_05_		8	10

**Table 3:** Biochemical composition of grain of the studied oat lines.

Line Number	Protein Content, %	Starch Content, %	Lysine Content, %	Lipid Content, %
Line no. 1, Bacha	14,7	57,5	7.4	8.8
Line no. 4	16.5	58.1	8.5	9.9
Line no. 7	16.9	61.0	6,5	7,0
Line no. 10	18.3	63.5	8.1	8.5
Line no. 13	19.7	64.0	9.4	9.0
Var. Ulov (chaffy)	12.8	51.3	3.8	4.5–5.2

## Data Availability

Not applicable.
